# KSL-POSE: A Real-Time 2D Human Pose Estimation Method Based on Modified YOLOv8-Pose Framework

**DOI:** 10.3390/s24196249

**Published:** 2024-09-26

**Authors:** Tianyi Lu, Ke Cheng, Xuecheng Hua, Suning Qin

**Affiliations:** School of Computer, Jiangsu University of Science and Technology, Zhenjiang 212100, China; chnlty671@gmail.com (T.L.); huaxuecheng8888@gmail.com (X.H.); 18118814562@163.com (S.Q.)

**Keywords:** human pose estimation, Kolmogorov–Arnold networks, YOLOv8-pose, multi-scale feature fusion

## Abstract

Two-dimensional human pose estimation aims to equip computers with the ability to accurately recognize human keypoints and comprehend their spatial contexts within media content. However, the accuracy of real-time human pose estimation diminishes when processing images with occluded body parts or overlapped individuals. To address these issues, we propose a method based on the YOLO framework. We integrate the convolutional concepts of Kolmogorov–Arnold Networks (KANs) through introducing non-linear activation functions to enhance the feature extraction capabilities of the convolutional kernels. Moreover, to improve the detection of small target keypoints, we integrate the cross-stage partial (CSP) approach and utilize the small object enhance pyramid (SOEP) module for feature integration. We also innovatively incorporate a layered shared convolution with batch normalization detection head (LSCB), consisting of multiple shared convolutional layers and batch normalization layers, to enable cross-stage feature fusion and address the low utilization of model parameters. Given the structure and purpose of the proposed model, we name it KSL-POSE. Compared to the baseline model YOLOv8l-POSE, KSL-POSE achieves significant improvements, increasing the average detection accuracy by 1.5% on the public MS COCO 2017 data set. Furthermore, the model also demonstrates competitive performance on the CrowdPOSE data set, thus validating its generalization ability.

## 1. Introduction

Human pose estimation is a vital research area in the computer vision field, which is focused on accurately identifying the spatial positions of key human joints in media content. Real-time human pose recognition is essential in various fields, including motion tracking, gesture recognition, motion analysis, and intelligent surveillance systems. It has extensive applications in virtual reality, behavior recognition, sports training, and the film and animation industries [1,2]. Achieving accurate 2D human pose estimation allows for optimization of model architectures and feature learning, thereby enhancing the precision and efficiency of pose estimation, ultimately leading to safer and more efficient services.

**Figure 1:**
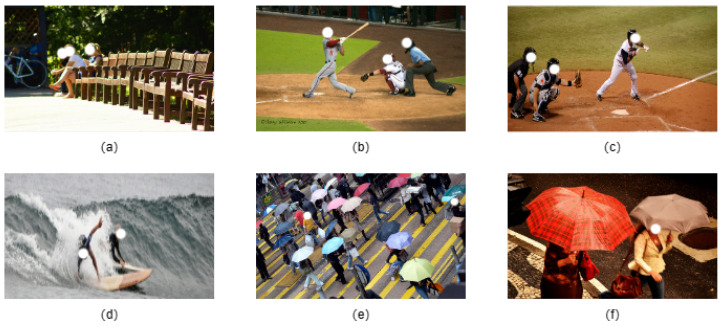
(**a**–**c**) demonstrate varying degrees of joint deformation that occur during human activities; (**d**–**f**) highlight instances where body parts are obscured either by objects or the individual.

There are two types of tasks in the human pose recognition field: single-person pose estimation and multi-person pose estimation. In single-person pose estimation, the model focuses on identifying and regressing the keypoint information of the target person, which includes keypoint categories, the relative positions of keypoints, and confidence levels. In contrast, multi-person pose estimation involves simultaneously detecting and estimating the keypoint information of multiple individuals. In these tasks, various degrees of joint deformation occur during human activities (as illustrated in Figure 1a–c), and body parts may be occluded by other objects or the individuals themselves (as shown in Figure 1d–f). These factors can lead to the pose estimation algorithm either failing to obtain keypoint information or only capturing a small amount, ultimately resulting in poor pose estimation outcomes. Therefore, this research aims to better address the challenges of capturing subtle and complex features, regressing occluded keypoints, and detecting small targets in human pose estimation tasks.

In recent years, with the continuous development of Deep Neural Networks (DNNs) [3], Convolutional Neural Networks (CNNs) [4], Recurrent Neural Networks (RNNs) [5], attention mechanisms [6], Transformers [7], and Kolmogorov–Arnold Networks (KANs) [8], researchers have significantly enhanced the regression capability of models for keypoints. They have achieved this through leveraging well-trained network architectures, making structural modifications, and applying post-optimization processes. Moreover, various strategies have been adopted to reduce the inference cost of networks, such as lightweight network design [9,10], spatial pyramid pooling [11], and other techniques. Researchers have also endeavored to integrate human pose estimation with object detection to address a broader range of practical scenarios [12]. For example, Ghiasi et al. have proposed NAS-FPN [13], which uses neural network structure search to discover a new feature pyramid structure in a new scalable search space covering all cross-scale connections and can fuse the input features of any two layers into the output features of one layer.

The YOLO series models [14], which are popular in the field of computer vision, were designed to predict the locations and categories of objects in an image through single-stage propagation of the network, significantly improving their detection speed and efficiency. YOLOv8, an advanced version of this series, has made several improvements in network structure and training strategies, resulting in exceptional performance in both object detection and pose estimation tasks. YOLOv8 introduces new feature extraction modules and more efficient anchor box designs, greatly enhancing the model’s detection accuracy and speed. It supports multi-scale fusion and employs spatial pyramid pooling and cross-stage partial networks for feature fusion, effectively capturing fine details in images. Furthermore, YOLOv8 integrates an efficient aggregation network and adopts more effective loss functions and optimization strategies, enabling the model to converge faster during training and achieve considerable detection accuracy. Despite numerous researchers having continuously improved or proposed highly effective human pose estimation models, the field still faces challenges such as partial joint loss due to arbitrary occlusion or complex backgrounds and difficulties in detecting small targets. Given the aforementioned challenges, we propose the KSL-POSE model based on YOLOv8l-POSE and inspired by the YOLO-POSE real-time framework. Through incorporating KAN convolutions, we enhance the model’s feature extraction capabilities. Moreover, we introduce the SOEP module and a self-developed detection head, which help to improve its pose estimation performance for small target detection, significantly enhancing the model’s performance in complex scenarios.

Overall, the contributions of KSL-POSE are as follows:

- First, KSL-POSE improves the backbone of the YOLOv8l-POSE baseline. We integrate the C2f feature extraction module with the KAN network, introducing non-linear activation functions to enhance the feature extraction capability of the convolutional kernels.
- Second, we introduce the small object enhance pyramid (SOEP) module, leveraging the cross-stage partial (CSP) [15] concept to further optimize and enhance the detail feature extraction performance on small targets.
- Finally, KSL-POSE combines the NAS-FPN concept and introduces a novel shared convolutional detection head. Unlike NAS-FPN, this detection module is composed of multiple shared convolutional layers and batch normalization (BN) layers, where BN is calculated independently, thus improving the utilization efficiency of model parameters and the cross-stage feature fusion capability.

## 2. Related Work

### 2.1. Top-Down Methods

Top-down human pose estimation methods involve locating instances through rectangular bounding boxes, then cropping these instances to obtain and detect keypoints within them. Representative algorithms of this type include CPN, HRNet, and Hourglass. Despite having specific applications in human pose estimation, top-down methods are limited in practical use due to their high computational complexity, poor real-time performance, strong dependency on bounding boxes, difficulty in handling multiple targets, and insufficient utilization of contextual information. For example, handling multi-person scenarios is challenging, especially when multiple bodies occlude or overlap each other. As the keypoint localization process within each bounding box is usually independent, the model cannot fully leverage global contextual information, leading to sub-optimal performance in both bounding box detection and keypoint localization.

### 2.2. Bottom-Up Methods

Bottom-up methods first detect all possible keypoints, then combine these keypoints into complete human poses through the use of clustering or matching techniques. The main characteristic of these methods is building the overall pose from local features (keypoints) incrementally. Representative methods include OpenPOSE, PersonLab, and HigherHRNet. While bottom-up methods are relatively effective in handling multi-person scenarios, their practical application is limited by their complex post-processing, difficulty in keypoint association, high computational burden, insufficient robustness, and reliance on global consistency.

Researchers have made various improvements and innovations in the field of human pose estimation. Zhang et al. [16] have proposed HF-HRNet, which enables enhanced multi-scale spatial feature fusion through the use of a cascaded depth (CAD) module and multi-scale context embedding (MCE) module, resulting in notable improvements in inference speed and model accuracy. Yang et al. [17] have introduced TransPOSE, which leverages Transformers and the self-attention mechanism to provide a similarity measure space between positional pairs, enabling the correct association of keypoints to corresponding human instances and effectively capturing long-term relationships. Qiu et al. [18] have proposed DiffusionPOSE, a model that significantly improves the accuracy of identifying human keypoints through utilization of noise heatmap generation, a progressive denoising process, conditional supervision based on human structural information, and an efficient architectural design. Liu et al. [19] have introduced LDCNet, which incorporates limb direction cues (LDCs) to suppress uncertainties in keypoint positions, thus preventing deep networks from overfitting uncertain keypoint locations. Moreover, researchers have experimented with incorporating attention mechanisms into networks, such as LSKA [20], DAttention [21], CA [22], and SA [23]. Notably, DAttention introduces a deformable self-attention module that selects the positions of keys and values in a data-dependent manner, allowing the model to focus more effectively on relevant areas and reduce the influence of irrelevant features.

The aforementioned algorithms have been shown to provide good inference efficiency and competitive detection accuracy in the field of human pose estimation. However, they still have shortcomings in addressing small target detection and arbitrary occlusion problems. Therefore, it is necessary to develop a human pose estimation method that can effectively address these issues.

## 3. Method

### 3.1. Overview

**Figure 2:**
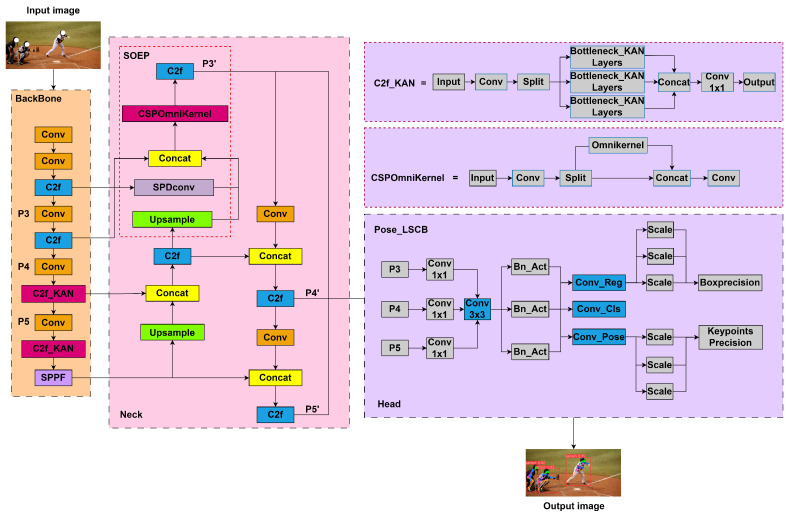
The overall framework of the KSL-POSE model.

The KSL-POSE model builds upon the YOLOv8l-POSE real-time framework for human pose estimation. Specifically, we draw inspiration from the recent advancements in Kolmogorov–Arnold Networks (KANs) and incorporate non-linear activation functions based on the C2f module, which we refer to as the C2f_KAN module. Moreover, we introduce the small object enhance pyramid (SOEP) module, which processes feature layers through SPDConv to extract feature information enriched with small object details, and integrates this information into the corresponding feature layers. We also incorporate a custom layered shared convolution with batch normalization detection head (LSCB). This design improves the accuracy and efficiency of keypoint detection through multi-scale feature fusion and shared convolutional layers. Through end-to-end training, KSL-POSE can accurately localize human regions and regress keypoints in real-time for each body part in an image. The overall framework is illustrated in Figure 2.

### 3.2. C2f_KAN Module

**Figure 3:**
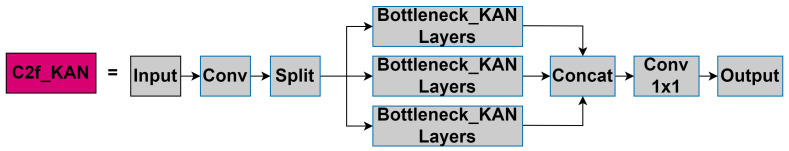
C2f_KAN module diagram.

In human pose estimation tasks, the use of standard convolution modules may lead to insufficient utilization of computational resources due to a large number of redundant features, as well as internal covariate shift issues during the training process, which can result in inadequate image feature extraction. To address these problems, we introduce the C2f_KAN module into the backbone part. This module incorporates the idea of instance normalization and has significant advantages in feature extraction and information fusion, making it particularly suitable for handling fine-grained features in human pose estimation. Its structure is shown in Figure 3.

The introduction of the KAN caused a sensation. In contrast to traditional MLPs, which use fixed activation functions at the nodes (“neurons”), a KAN implements learnable activation functions along the edges (“weights”). Specifically, the weight parameters in a KAN are replaced by univariate functions parameterized as spline curves, thereby eliminating linear weights. This adjustment allows KANs to excel in small-scale AI and scientific tasks, obtaining advances in both accuracy and interpretability. The underlying mathematical principle can be written as follows:

(1) $$f\left(x\right)=f\left(x_{1},\cdots ,x_{n}\right)=\sum_{q=1}^{2n+1}\Phi_{q}\left(\sum_{p=1}^{n}\phi_{q,p}\left(x_{p}\right)\right),$$

where $\Phi_{q,p}:\left[0,1\right]\to R$ and $\Phi_{q}:R\to R$.

To optimize computation, KAN convolution incorporates a basis function, b(x), in the design of the activation function. Therefore, the activation function ϕ(x) is the sum of the basis function b(x) and the spline function spline(x). Specifically, their mathematical formulas can be written as follows:

(2) ϕ(x)=w(b(x)+spline(x)),

(3) $$b\left(x\right)=silu\left(x\right)=x/\left(1+e^{-x}\right),$$

(4) $$spline\left(x\right)=\sum_{i}c_{i}B_{i}\left(x\right),$$

where ω is a weight that can better control the overall size of the activation function, $c_{i}$ denotes the coefficients that are optimized during training, $B_{i}\left(x\right)$ is a B-spline basis function defined on the grid, and the grid points define the interval where each basis function $B_{i}\left(x\right)$ is active, significantly affecting the shape and smoothness. During training, the parameters $c_{i}$ of these splines (i.e., the coefficients of the basis function $B_{i}\left(x\right)$) are optimized to minimize the loss function, thereby adjusting the shape of the spline to best fit the training data.

The C2f_KAN module, as shown in Algorithms 1–3, inspired by the KAN, retains the efficient feature extraction capabilities of the original C2f module while significantly enhancing its feature extraction and adaptability. Through integrating KAN convolution and non-linear activation functions, this module strengthens the convolutional kernels, enabling them to capture more intricate and subtle features. The C2f_KAN module is utilized in the P4 and P5 layers of the backbone network.

To balance parameter reduction with feature extraction performance, multiple Bottleneck_KAN modules are used to progressively extract and refine features, ensuring that the final output feature map is both rich and high in quality. Moreover, during the concatenation of features from different layers, 1×1 convolution is applied for dimensionality reduction and fusion, ensuring that the feature map’s dimensions and information content are optimal for further processing. The convolution function in the C2f_KAN module can be written as follows:

(5) $$y_{i,j,k}=\sum_{m=1}^{M}\sum_{n=1}^{N}x_{i+m-1,j+n-1,l}\cdot w_{m,n,l,k}+b_{k},$$

where *x* represents the input feature map with dimensions H×W×C (*H* is the height, *W* is the width, and *C* is the number of channels), ω is the convolution kernel with dimensions M×N×C×K (*M* is the kernel height, *N* is the kernel width, *C* is the number of input channels, and *K* is the number of output channels), $b_{k}$ (corresponding to the output channels *K*) denotes the bias term, and $y_{i,j,k}$ is the output feature map (where *i*, *j*, and *k* are the indices for the height, width, and channel dimensions of the output feature map, respectively) with dimensions $H^{\prime }\times W^{\prime }\times K^{\prime }$ ($H^{\prime }$ and $W^{\prime }$ are the height and width of the output feature map, and $K^{\prime }$ is the number of output channels)
**Algorithm 1** PyTorch-like Code for C2f_KAN Algorithm1:**Input:** c1,c2,n,kan_method,shortcut,g,e2:**Output:** Output tensor after processing through all Bottleneck_KAN layers3:Initialize: super()4:Create a list of Bottleneck_KAN modules: self.m ←$\mathrm{nn}.\mathrm{ModuleList}(\mathrm{Bottleneck}\text{\_}\mathrm{KAN}\left(c1,c2,kan\text{\_}method,shortcut,g,k=\left(3,3\right),e=\;1.0\right)\;{\mathrm{for}\;}_{\mathrm{in}\;\mathrm{range}}\left(n\right))$5:**for** each layer in self.m **do**6:   x←layer(x)7:**end for**8:**Return***x*

**Algorithm 2** PyTorch-like Code for Bottleneck_KAN Module
**Input:** c1,c2,kan_method,shortcut,g,k,e
2:**Output:** Output tensor after applying bottleneck operationsInitialize: super()4:Compute: $c_{\leftarrow }\mathrm{int}\left(c2\times e\right)$Define: $\mathrm{self}.\mathrm{cv}1\leftarrow \mathrm{choose}\text{\_}\mathrm{kan}(kan\text{\_}method,c1,c_{,}k\left[0\right])$6:Define: $\mathrm{self}.\mathrm{cv}2\leftarrow \mathrm{choose}\text{\_}\mathrm{kan}(kan\text{\_}method,c_{,}c2,k\left[1\right])$Compute: self.add←shortcutandc1==c28:**if** not self.add **then**   **Return** self.cv2(self.cv1(x))10:
**else**
  **Return** x+self.cv2(self.cv1(x))12:
**end if**



**Algorithm 3** PyTorch-like Code for choose_kan Function
**Input:** name,c1,c2,k**Output:** Selected KAN convolutional layer
3:
**if**  name==’FastKANConv2DLayer’  **then**
   kan←FastKANConv2DLayer(c1,c2,kernel_size=k,padding=k//2)
**else if**  name==’KANConv2DLayer’  **then**
6:   kan←KANConv2DLayer(c1,c2,kernel_size=k,padding=k//2)
**else if**  name==’KALNConv2DLayer’  **then**
   kan←KALNConv2DLayer(c1,c2,kernel_size=k,padding=k//2)9:
**else if**  name==’KACNConv2DLayer’  **then**
   kan←KACNConv2DLayer(c1,c2,kernel_size=k,padding=k//2)
**else if**  name==’KAGNConv2DLayer’  **then**
12: kan←KAGNConv2DLayer(c1,c2,kernel_size=k,padding=k//2)
**end if**
**Return** kan


### 3.3. SOEP Module

**Figure 4:**
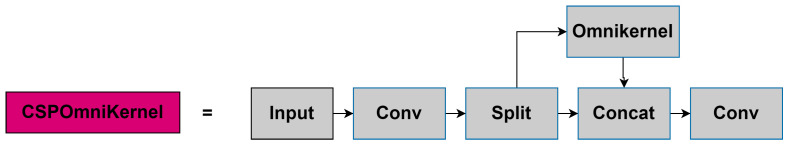
CSP-OmniKernel module diagram.

Detecting small targets during human pose estimation on the P3, P4, and P5 layers can be challenging. Traditional methods often add an extra detection layer before the P3 layer to enhance the detection of small targets. However, this can lead to increased computational complexity and longer post-processing times. To address these challenges, we improved upon the CSP approach and OmniKernel [24] through creating the CSPOmniKernel, which we refer to as the small object enhance pyramid (SOEP). We apply depthwise convolutions with kernel size of K×K to pursue a large receptive field and use 1×K and K×1 depth convolutions to obtain context information. To avoid introducing a large amount of computational overhead, we place the module in the neck part. Then, we explore the possibility of using maximal convolutions for image restoration by gradually increasing *k*, and reasonably expand the convolution kernel (receptive field) to the feature size while avoiding a large number of parameters. Instead of adding a detection layer, we process the data through SPDConv, which extracts features enriched with small target information. These features are then integrated into the P3 layer. This approach effectively learns feature representations from global to local scales, ultimately improving the detection performance for small targets. The structure of this method is illustrated in Figure 4.

### 3.4. LSCB Module

**Figure 5:**
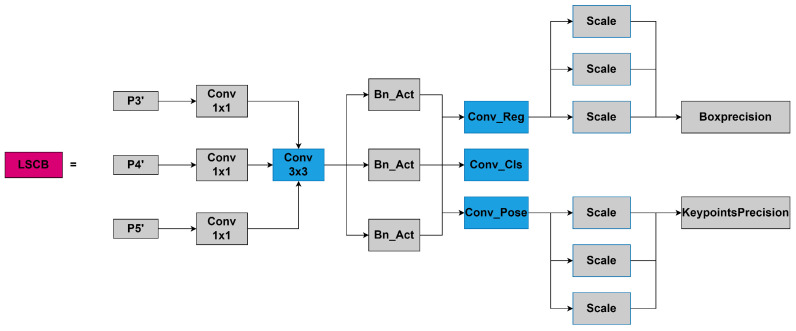
LSCB module diagram. Shared convolutional layers are filled in blue.

The layered shared convolution with batch normalization (LSCB) detection head is designed to receive feature maps from different scales (P3, P4, P5) using a multi-scale feature fusion method. By merging multi-scale features, it processes the feature maps from different scales to include information of varying granularity, thus capturing more contextual information. This helps the model to more accurately locate keypoints. Moreover, the LSCB module achieves the goal of reducing the number of parameters and improving computational efficiency through the use of shared convolutional layers, ensuring the real-time performance of the model. The resulting diagram is shown in Figure 5.

High-precision keypoint detection is essential for human pose estimation tasks. In the LSCB module, the keypoint feature processing layers consist of a 1×1 convolution layer, a 3×3 convolution layer, and another 1×1 convolution layer. These layers are designed to handle feature maps at different scales and to extract keypoint features effectively. The LSCB module processes multi-scale (P3’, P4’, P5’) features utilizing shared convolution layers, thereby capturing more contextual information from various scales of feature maps. Given the statistical differences between features at different levels, normalization is necessary. Directly incorporating batch normalization (BN) in the shared parameter detection head can cause errors due to moving average discrepancies. While group normalization (GN) can mitigate this issue, it increases the computational overhead during inference. Therefore, we independently calculate BN for each shared convolution layer, avoiding the moving average errors associated with shared BN. This approach significantly reduces the number of parameters. Moreover, to handle the inconsistent target scales detected by each detection head, we employ a Scale layer to adjust the features, ensuring that each detection head can adapt to different target scales. Specifically, after the three feature layers output from the neck enter the detection head, each branch first passes through a 1×1 convolution layer to adjust the number of channels, unifying the number of channels of the three input feature layers to the number of channels in the middle layer. Then, all feature layers are gathered into a shared convolution module for feature extraction. The convolution kernel size is 3×3. The use of shared convolution can reduce the number of parameters and calculations of the model. Finally, the regression branch and the classification branch are separated. In the regression branch, a 1×1 convolution layer is used to predict the coordinate offset of the bounding box. However, when there are objects with significant differences in size and shape within a scene, shared convolution may not fully adapt to targets of varying scales and shapes, potentially leading to insufficient representational capacity of the feature map. To compensate for its limitations in integrating multi-scale features, architectures like Feature Pyramid Networks (FPNs) or Path Aggregation Networks (PANs) can be introduced on top of shared convolution. These techniques help the model capture features of different scales without significantly increasing the number of parameters. Specifically, in LSCB, to address the issue of inconsistent target scales detected by each detection head, the output of the regression branch utilizes the Scale layer to scale the features and adjust the target scale, thereby enabling the localization of targets of different sizes. A similar mechanism can be observed in architectures like FPN, where multi-scale feature fusion improves detection performance for objects of various sizes. The introduction of the Scale layer is a further optimization of existing multi-scale fusion methods, adding flexibility and controllability. This enhances the applicability of shared convolution in multi-scale target detection and mitigates its negative impact on the quality of feature maps.

In the classification branch, a 1×1 convolution layer is used to predict the probability of each category. The convolution layer weights of the two branches are independent, such that the model can learn the positioning and classification tasks separately. Finally, the predicted keypoint features are decoded into actual keypoint coordinates, ensuring the accuracy of keypoint locations and improving detection precision.

Overall, the shared weight parameter design can effectively reduce the number of parameters and calculations, thus improving the running speed of the model, and allows for the processing of features of different scales at the same time, capturing information of various sizes in the image, which helps the model to better understand the relationships between objects in the image, thus improving the recognition accuracy.

## 4. Experiment

We evaluated the proposed model on both the COCO-POSE 2017 data set and the CrowdPOSE data set. In addition, we conducted ablation experiments to analyze the effects of various modules. These experiments were conducted on the Ubuntu 22.04.4 LTS operating system, utilizing an NVIDIA RTX 3090 GPU for acceleration. The KSL-YOLO model was constructed based on Pytorch 2.3.1 and Cuda 11.5.

### 4.1. Data Sets

The COCO-POSE 2017 data set [14] is an invaluable and widely utilized resource that has significantly advanced human pose estimation technology. It comprises 250,000 images annotated with keypoints, where each human instance in an image is marked with 17 keypoints. These keypoints provide the coordinates of major human joints, with each keypoint accompanied by a visibility flag indicating its visibility in the image. This data set supports researchers and developers in creating and evaluating advanced computer vision algorithms, thereby facilitating various application scenarios.

The CrowdPOSE data set [25] was specifically designed for multi-person pose estimation tasks, addressing the unique challenges of pose estimation in densely populated scenes. Developed by researchers from the University of Science and Technology of China, Peking University, and SenseTime, this data set serves as a benchmark for high-quality pose estimation in crowded environments. The CrowdPOSE data set consists of 20,000 images, with each human instance annotated with 14 keypoints. It encompasses complex scenarios such as overlapping human instances, partial or complete occlusion by other individuals or objects, and varying degrees of joint deformation. This data set provides a comprehensive and challenging benchmark for pose estimation in dense crowds.

### 4.2. Evaluation Metrics and Training Configuration

To evaluate the model’s performance, this study primarily compares the average precision (AP) and average recall (AR) based on different Object Keypoint Similarity (OKS) thresholds; for example, the average precision at an OKS threshold of 50% (AP50), average recall at an OKS threshold of 50% (AR50), and mean average precision (mAP50-95) across all OKS thresholds from 50% to 95% (in 5% increments). These metrics allowed for a comprehensive evaluation of the model’s accuracy and robustness in detecting keypoints across varying degrees of precision and recall.

KSL-POSE is based on the YOLOv8l-POSE framework, and does not use Automatic Mixed Precision (AMP) training. The model uses the Adam optimizer, with a batch size of 16, an input image size of 640×640, an initial learning rate of 0.03, and is trained for 350 epochs.

### 4.3. Evaluation Results

We compared KSL-POSE with other models on the COCO-POSE 2017 data set, as detailed in Table 1.

**Figure 6:**
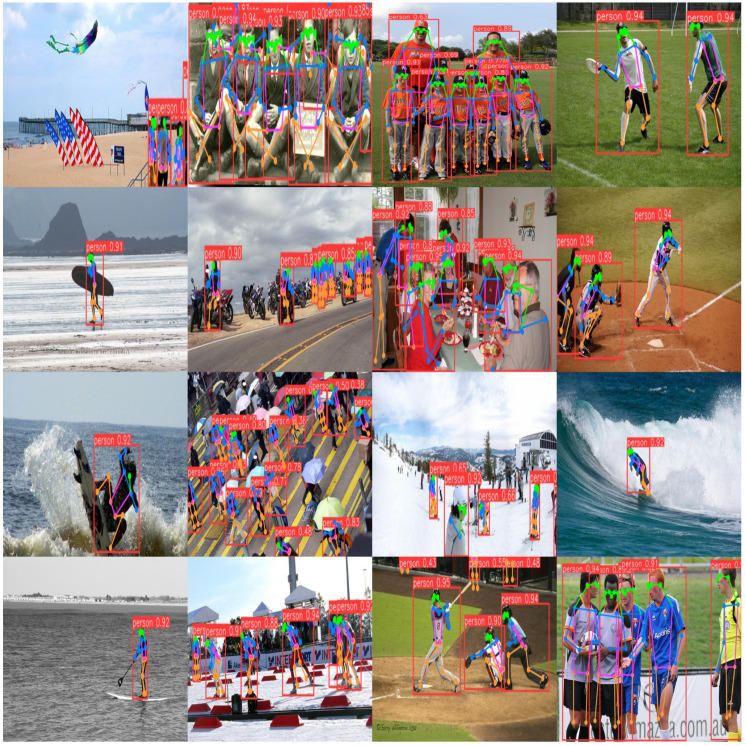
Results on the COCO-POSE 2017-val data set. The red boxes depict pedestrians of varying scales and the content in red boxes represent the accuracy of recognition, we have marked the keypoints with different colors.

The human pose estimation performance of KSL-POSE on the COCO-POSE 2017-val data set is shown in Figure 6.

Moreover, we also conducted comparisons on the CrowdPOSE data set, and the results are detailed in Table 2.

**Figure 7:**
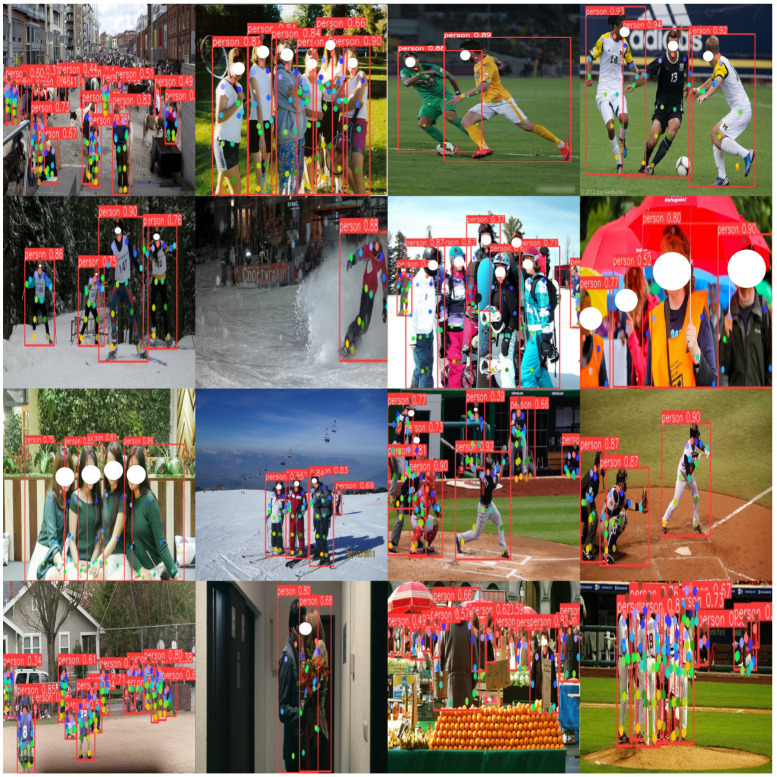
Results on the CrowdPOSE-val data set. The red boxes depict pedestrians of varying scales and the content in red boxes represent the accuracy of recognition; we have marked the keypoints with different colors.

The human pose estimation performance of KSL-POSE on the CrowdPOSE-val data set is shown in Figure 7.

The experimental results show that KSL-POSE improved the average precision on the COCO-POSE 2017-val set by 1.5% and AR by 2.2%, compared to the baseline YOLOV8l-POSE. The overall performance was not significantly different from that of YOLOv8l-POSE. Meanwhile, it also demonstrated competitive performance on the CrowdPOSE data set.

KSL-POSE performed well on both the COCO-POSE 2017-val and CrowdPOSE-val data sets, with its human pose estimation results shown in Figure 6 and Figure 7, respectively. When dealing with scenarios such as dense crowds, occluded body parts, and individual intersections, KSL-POSE maintained the accuracy of pose estimation. Moreover, it accurately identified and estimated the poses of most small targets, regardless of their relative positions in the image.

In summary, KSL-POSE presented good adaptability, robustness, and generalization in various practical application scenarios.

### 4.4. Ablation Experiments

To further evaluate the contribution of each module within the overall network structure, we conducted ablation experiments to assess the impact of each module on the entire network. We evaluated the effectiveness of each module through comparing the AP, AP50, and AP75. The ablation experiments were conducted on the COCO-POSE 2017-val set, and the specific experimental results are detailed in Table 3.

The ablation experiments indicated that KSL-POSE, compared to the baseline model, improved the average detection accuracy while only increasing the processing time by 30.3 ms. Specifically, AP50 and AP75 were improved by 0.9% and 1.1%, respectively. With the addition of the LSCB detection head, we effectively reduced the Floating Point Operations (FLOPs) and improved the model’s computational speed. Although incorporating this module slightly decreases recognition accuracy, as shown in Table 3, a comparison between the methods “+KAN+SOEP+LSCB” and “+KAN+SOEP” reveals that we can reduce the FLOPs by 37.5B while only sacrificing 0.7% in accuracy. This enhances the model’s computational efficiency and speed. On the other hand, when the LSCB module is not included, compared to the baseline model YOLOv8l-POSE, although the recognition accuracy increases, the FLOPs increase by 67.8B, significantly reducing the model’s computational efficiency. Overall, KSL-POSE gained significantly enhanced human pose estimation performance through innovatively combining the efficient feature extraction module C2f_KAN, the detail feature enhancement module SOEP, and the refined keypoint detection head LSCB.

**Figure 8:**
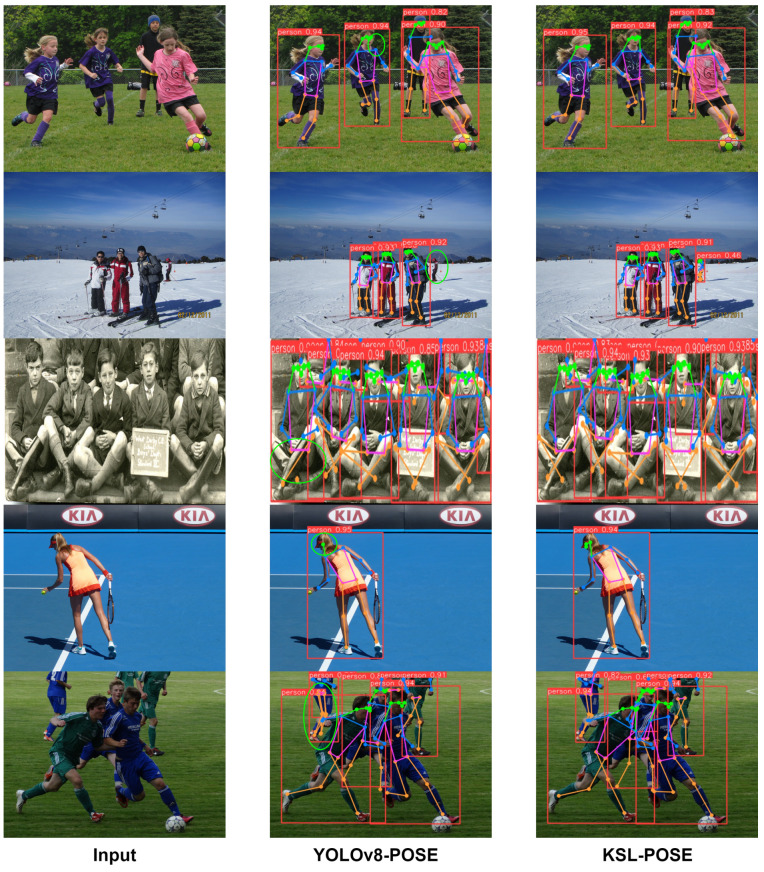
A visual comparison of YOLOv8l-POSE and KSL-POSE, with differences highlighted in green circles.

Moreover, we visualize the results of the YOLOv8l-POSE and KSL-POSE methods in Figure 8, highlighting the areas where the former failed to accurately recognize keypoints with green circles, while the red boxes depict pedestrians of varying scales and the content in red boxes represent the accuracy of recognition. YOLOv8l-POSE could not accurately identify keypoints or small targets when processing images with occluded body parts and overlapped individuals. However, through small target monitoring and shared convolutional layer methods, KSL-POSE can regress human pose positioning and corresponding skeletons in real-time and achieve precise and accurate recognition. The obtained results demonstrate that KSL-POSE provides an effective solution for human pose estimation.

## 5. Conclusions

In this study, we proposed an enhanced human pose estimation model, called KSL-POSE, based on YOLOv8l-POSE. Compared with other pose estimation methods, we are more concerned with solving the problems that occur when keypoints are not prominent or occluded. Therefore, we drew inspiration from KAN and introduced non-linear activation functions and instance normalization ideas to handle fine-grained features in human pose estimation. As shown in Table 3, KSL-POSE increased the AP value of the model by 0.8%. In addition, we also introduced SOEP and LSCB modules to optimize the quality of the corresponding generated keypoints through small target detection, multi-scale feature fusion, and shared convolutional layer methods, in order to better adapt to local occlusion in the human pose recognition task and improve the model’s ability to determine human joint points. With these modifications, Table 3 demonstrates a 1.5% improvement in the model’s AP. Through introducing the C2f_KAN module, SOEP module, and POSE_LSCB detection head, KSL-POSE achieves significant improvements in both keypoint detection accuracy and computational efficiency.

KSL-POSE was thoroughly evaluated on the COCO-POSE 2017 and CrowdPOSE human pose estimation data sets. The results indicate that this model surpasses the existing YOLOv8l-POSE model, in terms of average precision (AP) and average recall (AR). Although KSL-POSE showed a slight increase in FLOPs, and increased model complexity, it can capture more feature information, thus performing better in handling occluded targets and small object detection. Overall, KSL-POSE can efficiently perform real-time human pose estimation tasks. Future research will focus on further optimizing the KSL-POSE model to make it more lightweight, aiming to reduce the number of parameters and computational cost while maintaining its accuracy, thereby enhancing its adaptability to various environments and tasks.

## Figures and Tables

**Table 1:** Comparison of various real-time pose estimation methods on the COCO-POSE 2017 data set.

Method	AP	AP50	AP75	APM	APL	AR
Hourglass [26]	63.0	85.7	68.9	58.0	70.4	-
HGG [27]	67.6	85.1	73.7	62.7	74.6	-
OpenPOSE [28]	61.8	84.9	67.5	57.1	68.2	66.5
HRNet	64.5	86.2	71.7	59.2	73.5	-
HigherHRNet [29]	70.5	89.6	76.9	65.1	76.9	-
YOLOv5m6-rlepose [30]	67.6	89.2	74.5	-	73.8	74.0
YOLOv8-PoseBoost [31]	-	85.4	59.1	60.1	74.2	-
YOLOv8l-POSE	67.6	90.0	74.6	63.3	75.7	74.9
SDPose-T [32]	69.2	90.2	76.8	65.7	75.2	-
YOLOv8x-POSE	71.7	91.5	78.4	68.2	78.3	77.9
Diffusion RegPose [33]	72.5	89.9	79.5	66.8	80.5	-
Ours	69.1	90.9	75.5	65.2	76.6	76.7

**Table 2:** Comparison of various real-time pose estimation methods on the CrowdPose data set.

Method	AP	AP50	AP75	APE	APM	APH
OpenPOSE	48.0	61.5	53.7	62.7	48.7	32.3
Mask-RCNN [34]	57.2	83.5	60.3	69.4	57.9	45.8
SDPose-S-V2 [32]	64.5	-	-	-	-	-
HrHRNet-W48 [29]	67.6	87.4	72.6	75.8	68.1	58.9
OpenPifPaf [35]	70.5	89.1	76.1	63.8	78.4	72.1
Diffusion RegPose [33]	72.7	91.1	79.3	79.3	73.3	64.9
ViTPOSE-B [36]	73.4	91.8	77.9	78.8	75.1	64.5
Ours	68.3	89.6	74.9	77.2	70.5	60.8

**Table 3:** Ablation study of the proposed real-time pose estimation method on the CrowdPose data set.

Method	AP	AP50	AP75	FLOPs
YOLOv8l-POSE	67.6	90.0	74.6	168.6
+KAN+SOEP+LSCB	69.1	90.9	75.5	198.9
+KAN+SOEP	69.8	91.1	76.1	236.4
+SOEP+LSCB	68.8	89.7	73.4	187.7
+KAN+LSCB	67.2	88.8	72.3	130.1
+KAN	68.4	89.6	73.8	168.6
+SOEP	70.2	91.2	75.9	225.4
+LSCB	68.7	89.4	71.6	117.3

## Data Availability

The data sets analyzed in the study are openly available in [CrowdPose] at https://github.com/Jeff-sjtu/CrowdPose.git (accessed on 15 March 2024) and [MS COCO] at https://cocodataset.org/ (accessed on 20 December 2023).

## Abbreviations

The following abbreviations are used in this manuscript:

MDPI	Multidisciplinary Digital Publishing Institute
DOAJ	Directory of open access journals
TLA	Three letter acronym
LD	Linear dichroism
